# 07 Predictor factors of hip involvement in juvenile idiopathic arthritis

**DOI:** 10.1093/rheumatology/keac496.003

**Published:** 2022-10-07

**Authors:** Ben Aissa Rania, Ferjani Hanene, Moalla Mariam, Ben Nessib Dorra, Triki Wafa, Maatallah Kaouther, Kaffel Dhia, Hamdi Wafa

**Affiliations:** Departement of Rheumatology, Mohamed Kassab Institute of Orthopedics, Tunis, Tunisia

## Abstract

**Background:**

Juvenile idiopathic arthritis (JIA) is one of the commonest rheumatic diseases in children. Hip involvement is a common problem in JIA patients and is associated with functional disability and poor outcomes. Intensive therapy is required to avoid joint replacement surgery. Little studied in the literature, the predictors of hip involvement are still unknown.

**Objectives:**

Our study aims to identify the clinical, biological characteristic of patients with hip involvement and determine the associated risk factors.

**Methods:**

A cross-sectional study including children with JIA according to the International League of Associations for Rheumatology (ILAR). The recorded data included sociodemographic features, disease characteristics (subtype disease, duration, and juvenile arthritis disease activity score (JADAS)) as well as treatment modalities. Regarding coxitis, we collected radiographs, ultrasound (US), and magnetic resonance imaging (MRI) of the hip when performed. Coxitis was defined by clinical (limited range of motion) and/or radiographic findings (destruction, synovitis, bone marrow oedema).

**Results:**

Thirty-five patients (20 females) with a median age of 12 years (5–18) and disease duration of 3 years (0.25–15) were recruited. The patient's distribution of JIA subtypes were oligoarticular (*n* = 13), enthesitis-related arthritis (*n* = 9), polyarticular (*n* = 4) (negative rheumatoid factor in 3 patients), undifferentiated (*n* = 4), psoriatic-arthritis (*n* = 4) and systemic-onset (*n* = 1). ESR and CRP median values were 15 mm/h (0–63) and 2 mg/l(0–47) respectively. Sixteen patients were under DMARDs (Methotrexate (*n* = 10), biological agent (*n* = 3), biological agent and methotrexate (*n* = 2), salazopyrine (*n* = 1)).

Sixteen patients (45.71%) developed coxitis (radiographic (*n* = 8), MRI (*n* = 5) and US findings (*n* = 3)) with eight (50%) presenting limited range of motion and 10 (62.5%) developing radiological evidence of hip damage. Hip involvement was associated with a longer disease duration (*p* = 0.051). JADAS score value of patients with coxitis was higher (Mean 8.35 *vs* 7.05) but not significantly (*p* = 0.565). Higher CRP and ESR values were found in patients with coxitis (mean 8.39 mg/l *vs* 6.83 mg/l, 22.29 mm/h *vs* 16.37 mm/h respectively) but not significantly (*p* = 0.718, *p* = 0.287 respectively). No associations were found between hip involvement and BMI (*p* = 0.233), age-onset (*p* = 0.496), JIA subtype (*p* = 0.509), nor sex (*p* = 0.767).

**Conclusion:**

Our study shows that long disease duration exposes to a higher risk of hip involvement in children with JIA. Active disease and biological inflammatory syndrome could be associated risk factors. Studies with larger sample sizes are needed to draw definite causal associations.

